# Sucrose Synthase and Fructokinase Are Required for Proper Meristematic and Vascular Development

**DOI:** 10.3390/plants11081035

**Published:** 2022-04-11

**Authors:** Nitsan Lugassi, Ofer Stein, Aiman Egbaria, Eduard Belausov, Hanita Zemach, Tal Arad, David Granot, Nir Carmi

**Affiliations:** 1 Institute of Plant Sciences, Agricultural Research Organization, The Volcani Center, Rishon LeZion 7505101, Israel; lugassin@gmail.com (N.L.); oferstein@gmail.com (O.S.); aimaneg@tauex.tau.ac.il (A.E.); eddy@volcani.agri.gov.il (E.B.); hanita@volcani.agri.gov.il (H.Z.); talarad@agri.gov.il (T.A.); granot@volcani.agri.gov.il (D.G.); 2 The Robert H. Smith Faculty of Agriculture, Food and Environment, The Institute of Plant Sciences and Genetics in Agriculture, The Hebrew University of Jerusalem, Rehovot 76100, Israel

**Keywords:** auxin, phyllotaxis, shoot apical meristem, sugar metabolism, trichomes density, vascular tissues development

## Abstract

Sucrose synthase (SuSy) and fructokinase (FRK) work together to control carbohydrate flux in sink tissues. SuSy cleaves sucrose into fructose and UDP-glucose; whereas FRK phosphorylates fructose. Previous results have shown that suppression of the *SUS1,3&4* genes by SUS-RNAi alters auxin transport in the shoot apical meristems of tomato plants and affects cotyledons and leaf structure; whereas antisense suppression of *FRK2* affects vascular development. To explore the joint developmental roles of SuSy and FRK, we crossed SUS-RNAi plants with *FRK2*-antisense plants to create double-mutant plants. The double-mutant plants exhibited novel phenotypes that were absent from the parent lines. About a third of the plants showed arrested shoot apical meristem around the transition to flowering and developed ectopic meristems. Use of the auxin reporter DR5::VENUS revealed a significantly reduced auxin response in the shoot apical meristems of the double-mutant, indicating that auxin levels were low. Altered inflorescence phyllotaxis and significant disorientation of vascular tissues were also observed. In addition, the fruits and the seeds of the double-mutant plants were very small and the seeds had very low germination rates. These results show that *SUS1,3&4* and *FRK2* enzymes are jointly essential for proper meristematic and vascular development, and for fruit and seed development.

## 1. Introduction

Sugars in plants are produced from the fixation of carbon dioxide via photosynthesis, which occurs primarily in the mesophyll cells of mature leaves. Sugars are an essential energy source, as well as a major carbon backbone for structural components. In addition, they may also act as signaling molecules to regulate many developmental processes throughout the plant life cycle [1,2,3]. Sucrose, which is produced in the leaf mesophyll cells from the products of photosynthesis, is the main sugar transported by the phloem to sink tissues in many plant species, including tomato (*Solanum lycopersicum*). Upon arriving at a sink tissue, sucrose must be cleaved into its hexose monomers before it can enter any metabolic pathway. Sucrose can be cleaved by invertase to glucose and fructose, or by sucrose synthase (SuSy) in the presence of UDP to fructose and UDP-glucose (UDP-G; [4]). To be metabolized, free glucose and fructose must then be phosphorylated by hexokinase (HXK) and fructokinase (FRK), respectively. Fructose may also be phosphorylated by HXK, but the affinity of FRK to fructose is two orders of magnitude higher than that of HXK, rendering FRKs the primary fructose-phosphorylating enzymes [5].

While the roles of HXK in glucose-sensing and the regulation of plant responses to glucose levels have been extensively studied [2], other enzymes, such as SuSy and FRK have drawn far less attention in terms of possible roles in plant development [6]. The roles of *SUS* and *FRK* genes have been studied separately. There are four *FRK* genes and six *SUS* genes in the tomato. Three of the *FRK* genes (*FRK1,2,4*) encode cytosolic enzymes, while the fourth (*FRK3*) encodes a plastidic FRK [7]. *FRK2* is the main FRK gene expressed in most tomato organs [8,9] and is the only tomato *FRK* gene whose suppression yields severe growth inhibition accompanied by reduced hydraulic conductivity and leaf wilting due to the disturbing development of xylem vessels [10,11]. In contrast, suppression of a single *SUS* gene did not yield any significant visible phenotypes, but co-suppression of three *SUS* genes (*SUS1*, *SUS3*, and *SUS4*) caused abnormal cotyledon morphology as early as the embryo stage, aberrations in leaf structure stemming from altered auxin transport, reduced fruit set, and lower seed weights [12].

SuSy and FRK enzymes work in concert to control sucrose cleavage in various sink tissues [10,13]. Both SuSy and FRK2 are inhibited by fructose and, therefore, it was suggested that SuSy and FRK2 may form a double-brake mechanism to avoid excess sucrose catabolism. That is when SuSy cleaves too much sucrose and the accumulated fructose inhibits the activity of both FRK2 and SuSy, which may limit the amount of sucrose diverted to vascular development [10,14]. Yet, the joint roles of SuSy and FRK2 have not been studied in the context of plant development. The aim of this work was to study the joint roles of FRK2 and SuSy using tomato plants in which FRK2 and the SUS1,3,and4 enzymes were all suppressed.

## 2. Materials and Methods

### 2.1. Plant Material and Growth Conditions

Experiments were performed on tomato plants (*Solanum lycopersicum*, cv. MP1) and MP1 transgenic lines or hybrid crosses thereof as described below. Unless otherwise noted, unmodified MP1 plants served as the control group in all experiments. Plants were grown in a soil mixture of 70% tuff and 30% peat (Shaham, Givat Ada, Israel) in a temperature-controlled greenhouse under natural light conditions.

### 2.2. Transgenic Lines

The generation of the SUS-RNAi line is described in Goren et al. (2017) [12]. The generation of the *FRK2*-antisense plants is described in Dai et al. (2002) [15]. For the sake of simplicity, the *FRK2*-antisense line is referred to as *frk* and the S1R4 line is referred to as *sus*.

### 2.3. Shoot Tip Gene-Expression Analysis

Lateral shoot tips from WT, *sus*, *frk,* and *sus/frk* plants were excised about 1 cm from the edge and all leaves and inflorescences that were visible to the naked eye were removed using forceps. Each sample was comprised of three lateral shoot tips. Six samples were collected from each line, including the WT. Samples were frozen immediately for total RNA extraction using the Logspin method [16]. In brief, samples were ground using a Geno/Grinder (SPEX SamplePrep, Metuchen, NJ, USA), and RNA was extracted in 8 M guanidine hydrochloride buffer (Duchefa Biochemie) and transferred to tubes containing 96% EtOH (Bio Lab, Jerusalem, Israel). Then, the samples were transferred through a plasmid DNA extraction column (RBC Bioscience, New Taipei City, Taiwan), followed by two washes in 3 M Na-acetate (BDH Chemicals, Mumbai, India) and two washes in 75% EtOH, and then an elution with DEPC (diethylpyrocarbonate) water (Biological Industries Co., Beit Haemek, Israel) that had been preheated to 65 °C. The RNA was treated with RQ1-DNase (ProMega, Madison, WI, USA) according to the manufacturer’s instructions, to degrade any residual DNA.

For the preparation of cDNA, total RNA (1 µg) was taken for reverse transcription-PCR using qScript™ cDNA Synthesis Kit (Quanta BioSciences, Gaithersburg, MD, USA) following the manufacturer’s instructions. cDNA samples were diluted 1:7 in double-distilled water. Quantitative real-time PCR reactions were performed using SYBR Green mix (Thermo-Scientific, Waltham, MA, USA) and reactions were run in a RotorGene 6000 cycler (Corbett, Mortlake, New South Wales, Australia). Following an initial pre-heating step at 95 °C for 15 min, there were 40 cycles of amplification each consisting of 10 s at 95 °C, 15 s at 55 °C, 10 s at 60 °C, and 20 s at 72 °C. Results were analyzed using the RotorGene software. Data were normalized using *cyclophilin* as a reference gene. The primers used for amplification of the SUS genes were as described in previous work [17]. SUS1 primers were (SlSUS1_F-CTGCTGAGTGAATGAAGGTC) and (SlSUS1_R-GATACTAAATGGAAATGAAACAC), SUS3 primers were (SlSUS3_F-GGTTTCTGTCTGATTGTTATCC) and (SlSUS3_R-ACAGAAGGGAAAAATGGCAAA) and SUS4 primers were (SlSUS4_F-AACGTGGAGCATACCACTCT) and (SlSUS4_R-CAGCATATTGGATCACTGATTTG). The FRK2 primers were (F-CTTTGCTCAAGGGAGGAGCA) and (R-AGGAGAAGTTACAAGGAAGCTG) and the cyclophilin primers were (F-CGTCGTGTTTGGACAAGTTG) and (R-CCGCAGTCAGCAATAACCA).

### 2.4. DR5::VENUS Reporter Crosses

The *frk* transgenic line (MP1 background ecotype) was crossed with a tomato line bearing the pDR5rev:3XVENUS-N7 construct (M82 background ecotype; [18]). The F1 progeny were self-crossed and F2 segregants were selected for *frk*/DR5::VENUS. A double-homozygous segregant was crossed with the SUS-RNAi line. The F1 progeny were self-crossed and F2 segregants were selected for *sus*/DR5::VENUS and *sus/frk*/DR5::VENUS. WT/DR5::VENUS segregant served as a control. Since The DR5::VENUS line was in the background of M82 while the *sus*, *frk,* and *sus/frk* lines are in the background of MP1 we generated several (at least 3) segregants of each homozygous line (*frk*/DR5::VENUS, *sus*/DR5::VENUS and *sus/frk*/DR5::VENUS) and the WT and confirmed that their meristematic auxin response was similar.

### 2.5. Confocal-Microscopy Imaging

Shoot apices of the different segregant lines of DR5::VENUS were collected and the meristems were visualized under a confocal microscope. Images were acquired using the Olympus IX81 inverted laser scanning confocal microscope (Fluoview500) equipped with a 488-nm argon-ion laser. VENUS protein was excited by 488-nm light and the emission was collected using a BA 505–525 filter. A BA 660 IF emission filter was used to observe chlorophyll autofluorescence. Confocal optical sections were obtained in increments of 0.5 to l μm. The images were color-coded yellow for VENUS and magenta for chlorophyll autofluorescence.

### 2.6. Measurements of Seed Weight and Seed Germination

For the measurements of average seed weight, three replicates of 50 seeds each from three different fruit collections of WT, *sus*, *frk,* and *sus/frk* plants were weighed. Results are presented as the means of the three replicates.

To measure seed germination, at least four replicates of 25 seeds per line were germinated in Petri plates containing wet cotton wool, which were kept in darkness at room temperature. The germination rate per replicate was recorded daily for 8 days.

### 2.7. Embryo Imaging

We mechanically separated the embryos of at least three seeds of each line (five WT seeds, four *sus* seeds, three *frk* seeds, and seven *sus*/*frk* seeds), after 24 h of soaking in water, under a binocular microscope. Digital images were taken using a CCD camera DC2000 (Leica, Wetzlar, Germany). ImageJ (http://rsb.info.nih.gov/ij/, accessed on 6 March 2019) software was used to analyze the distance between the root apical meristem and the shoot apical meristem (SAM) in the different samples.

### 2.8. Scanning Electron Microscopy (SEM)

For scanning electron microscopy (SEM), leaflets and meristems were fixed in 3.7% formaldehyde, 50% ethanol, and 5% acetic acid by vacuum infiltration for 30 min and an additional 8 h in the fixative. The samples were dehydrated in a graded ethanol series (50%, 70%, and 90%, 100%, 100%, 60 min each). Dehydrated tissues were critical-point dried in liquid CO_2_ in a Quorum K850 critical-point dryer (Quorum Technologies, East Sussex, UK), and sputter-coated with gold-palladium (QuorumSC7620 mini sputter coater). Images were taken with a JEOL JCM-6000 benchtop SEM device.

### 2.9. Trichome Density

At least four leaflets from different plants were analyzed for each line and from the control (abaxial or adaxial side). The trichomes within a field of 1 mm^2^ were counted for each replicate of each line. Trichome counting was carried out using the ImageJ (http://rsb.info.nih.gov/ij/, accessed on 21 December 2017) software.

### 2.10. Anatomical Examination

To study and analyze the anatomy of both the phloem and the xylem tissues, freehand cross-sections were taken from the mature stems and inflorescences of the different lines. The cross-sections were stained for a few seconds in 2% lacmoid (Polyscience, Warrington, PA, USA) in 96% ethanol and then rinsed in tap water for a few minutes [19]. Cross-sections were observed under transmitted white light. The lacmoid stained the lignin in the cell walls of xylem vessels and fibers marine blue.

### 2.11. Statistical Analysis

All statistical analyses were carried out using the JMP 5.0 software platform (SAS Institute, Cary, NC, USA).

## 3. Results

### 3.1. Creation of the SUS and FRK2 Co-Suppressing Tomato Plants

**Figure 1:**
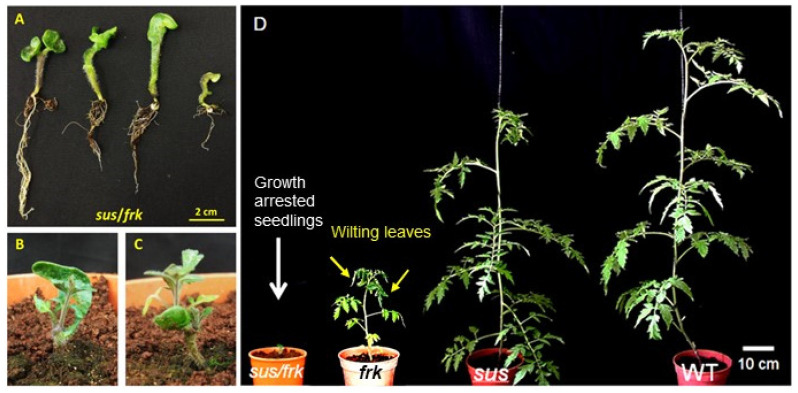
Phenotypes of F1 seedlings from the cross between the *sus* and *frk* plants. (**A**) Four of the F1 seedlings from the cross between the *sus* and *frk* plants at 8 weeks after germination. The seedlings did not show any visible shoot growth but did seem to have sufficient root systems. (**B**,**C**) Two seedlings from the same cross developed visible shoots, but remained abnormally short. (**D**) Six-week-old *sus*/*frk* growth-arrested seedling and *frk*, *sus,* and WT plants.

**Figure 2:**
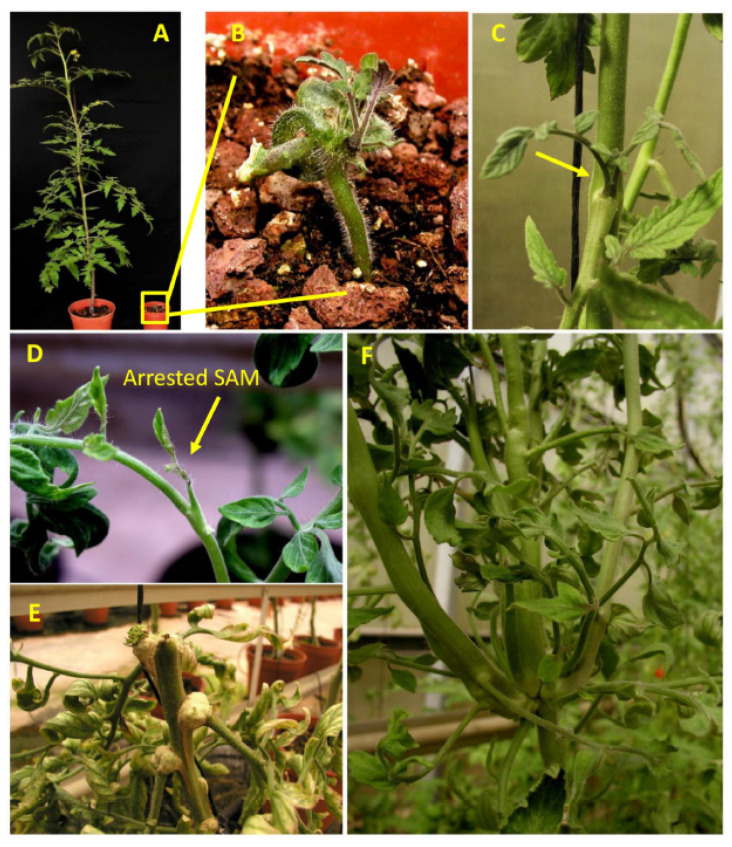
F2 segregating plants from the cross between *sus* and *frk* exhibited a variety of meristematic aberrations. (**A**) Eight-week-old WT (left) and F2 *sus/frk* segregant (right) whose growth was arrested at an early vegetative phase. (**B**) Enlarged image of the plant is shown in the yellow box in (**A**), indicating shoot apical meristem (SAM) growth arrest. (**C**) Two leaves (yellow arrow) developed from an auxiliary meristem of an F2 segregant instead of a lateral branch. (**D**) SAM growth arrest at the transition to flowering. (**E**) Callus tissue formed from the auxiliary meristems of an F2 *sus*/*frk* segregant that underwent SAM growth arrest. (**F**) Abnormal formation of several co-localized lateral branches of an F2 segregant.

To investigate the putative joint roles of SuSy and FRK2, we crossed the previously characterized *FRK2*-antisense line FK-3a,5 [10,11] and the SUS-RNAi line S1R4 [12] to obtain double-mutant lines. For the sake of simplicity, the *FRK2*-antisense line is referred to as *frk* and the S1R4 line is referred to as *sus*. Twenty seeds were obtained from two successful crosses, out of 10 crosses. Only nine out of the 20 seeds obtained from the cross germinated, and 55% of the seeds (11 out of twenty) were lethal. All nine F1 seedlings exhibited severely distorted cotyledons (Figure 1A), with distortion that was much more pronounced than that observed in the *sus* line (Figure S1; [12]). While the *sus* seedlings usually each had one abnormal cotyledon [12], in the F1 seedlings, either both cotyledons were distorted or fused, or one was missing and the other was distorted (Figure 1A and Figure S1). In addition, all nine plants exhibited severe growth inhibition, as compared to the *frk*, *sus,* and WT plants (Figure 1D). Eight weeks after germination, four of the seedlings did not show any visible shoot growth, but did seem to have sufficient root systems (Figure 1A); two seedlings had developed visible shoots but remained abnormally short (Figure 1B,C) and only two seedlings had managed to grow. Of the seedlings, ~78% failed to develop and produce flowers and seeds. Those two seedlings were planted in pots and eventually produced seeds that were sown to yield segregating F2 plants. Several of the F2 segregating plants exhibited a variety of meristematic aberrations including SAM growth arrest at an early vegetative phase (Figure 2A,B) or around the transition to flowering (Figure 2D), a transition that usually happens after the plant has about 11 leaves. In addition, abnormal formation of lateral branches (Figure 2C,F) and the formation of callus tissue from auxiliary meristems (Figure 2E) were also observed. Neither of these aberrations was observed in the single *sus* or *frk* mutants. Few of the double-mutant plants produced fruits with seeds that yielded F3 plants. We examined those few F3 plants, confirmed their homozygosity for both transgenes (*FRK2*-antisense and *SUS-RNAi*), and referred to them as *sus*/*frk*.

### 3.2. The sus/frk Homozygous Plants Exhibited Decreased Suppression of FRK2

**Figure 3:**
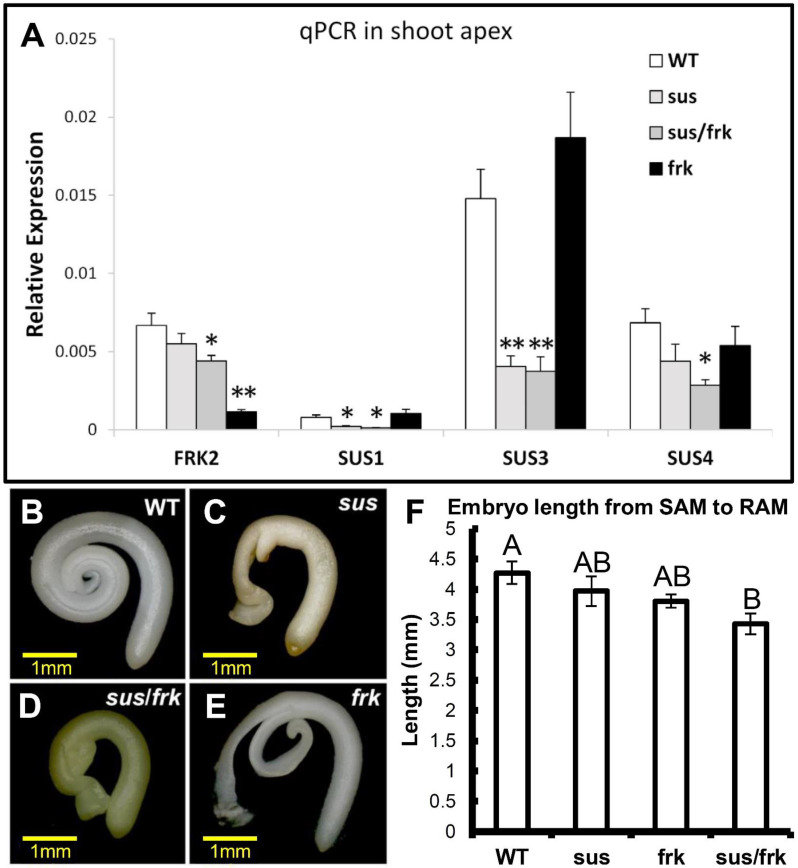
Expression of *SUS1,3,4* and *FRK2* in *sus*, *frk* and *sus/frk* homozygote plants and the effects of those mutations on embryo morphology. (**A**) The expression of *FRK2* and *SUS1,3&4* in *sus*, *frk*, *sus*/*frk* and WT plants was analyzed in RNA extracted from shoot apices. The suppression of the *SUS1* and *SUS3* genes was similar to that observed among the *sus* parent plants and the suppression of the *SUS4* gene was even stronger. Yet, the suppression of *FRK2* in *sus*/*frk* was significantly less than that observed for the *frk* parent. Error bars indicate the standard error (*n* = 6). Asterisks indicate a statistically significant difference relative to the WT (* *p* < 0.05; ** *p* < 0.01). (**B**–**E**) Embryos isolated from mature seeds of (**B**) WT, (**C**) *sus*, (**D**) *sus/frk* and (**E**) *frk*. (**F**) Embryo length from the SAM to the root apical meristem (RAM). Error bars indicate the standard error (*n*_WT_ = 5; *n*_sus_ = 5; *n*_frk_ = 7; n_sus/frk_ = 7). Different letters indicate a significant difference (*t*-test, *p* < 0.01).

**Figure 4:**
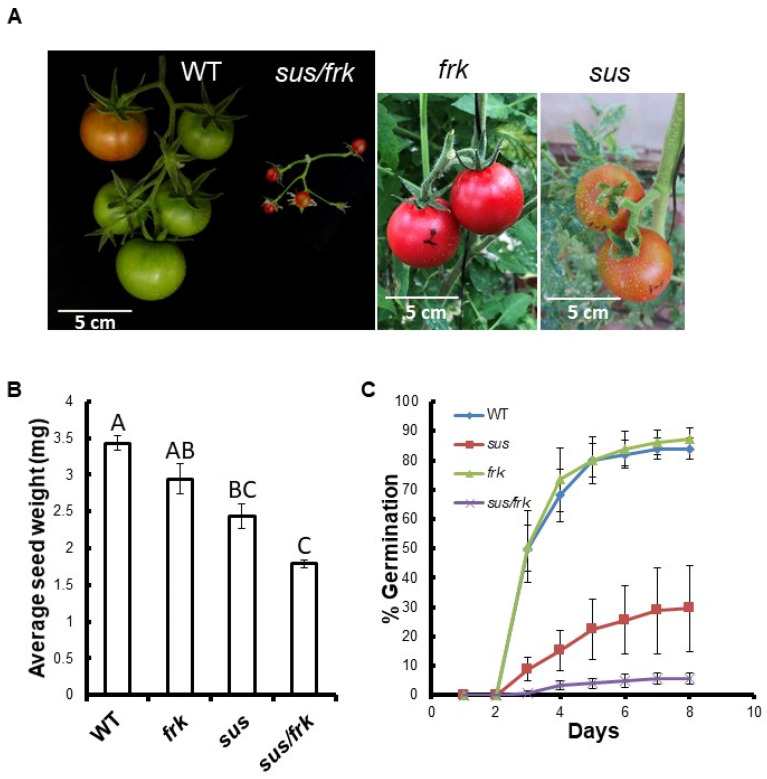
*sus*/*frk* plants have small fruits and small seeds with reduced germination rates. **(A**) From left to right: WT fruits, *sus*/*frk* fruits, *frk* fruits, and *sus* fruits. (**B**) Average seed weights for the WT, *frk*, *sus* and *sus*/*frk* (*n* = 3 × 50). (**C**) Germination rates of WT, *sus*, *frk* and *sus*/*frk* seeds (*n* ≥ 4 × 25). Error bars indicate the standard error. Different letters indicate a significant difference (*t*-test, *p* < 0.01).

The *SUS1,3,4* and *FRK2* genes were previously shown to be repressed in *sus* and *frk* plants, respectively, and we analyzed the expression levels of these genes in the *sus*/*frk* (homozygote) plants. The suppression of the *SUS1* and *SUS3* genes in the *sus*/*frk* plants was similar to that observed in the *sus* parent plants, with even stronger suppression of *SUS4* expression in the *sus*/*frk* plants (Figure 3A). Yet, the suppression of *FRK2* in the *sus*/*frk* plants was significantly less than that observed in the *frk* parent plant: 34% compared to 83%, respectively (Figure 3A). Accordingly, the visible phenotypes of the *sus*/*frk* homozygous plants were similar to that of the original *sus* parent plants (e.g., distorted cotyledons), but the *sus*/*frk* homozygous plants did not exhibit the severe growth inhibition and leaf wilting observed among the *frk* parent plants [10]. In fact, *sus*/*frk* homozygous plants grew just as tall as the WT and *sus* plants. The distorted cotyledons of the *sus*/*frk* seedlings could be observed at the embryo stage within the seed, similar to the *sus* parent plants (Figure 3B–E). Yet, the embryonic hypocotyls that developed from the SAM and the root meristem were slightly shorter than those of the *frk* and *sus* lines, and significantly shorter than those of the WT, suggesting an additive effect of the two transgenes (Figure 3F). In addition, while the fruits of the WT, *sus,* and *frk* lines were all similar in size, most of the fruits of the *sus*/*frk* plants were much smaller, varying from 1 to 2 cm in diameter, with no seeds (Figure 4A). Yet, we did manage to collect seeds from some of the larger fruits and examined their weight and germination. No significant reduction in seed weight was observed in the *frk* line, a 30% reduction was observed in the *sus* line and a 50% reduction in seed weight was observed in *sus*/*frk* line (Figure 4B). The *sus*/*frk* seeds had a very low germination rate of 6%, compared to 30% for the *sus* seeds and 84% for the WT and *frk* seeds (Figure 4C).

### 3.3. The sus/frk Plants Generated Calluses on the Adaxial Surfaces of Their Leaves and Exhibited Altered Inflorescence Architecture

**Figure 5:**
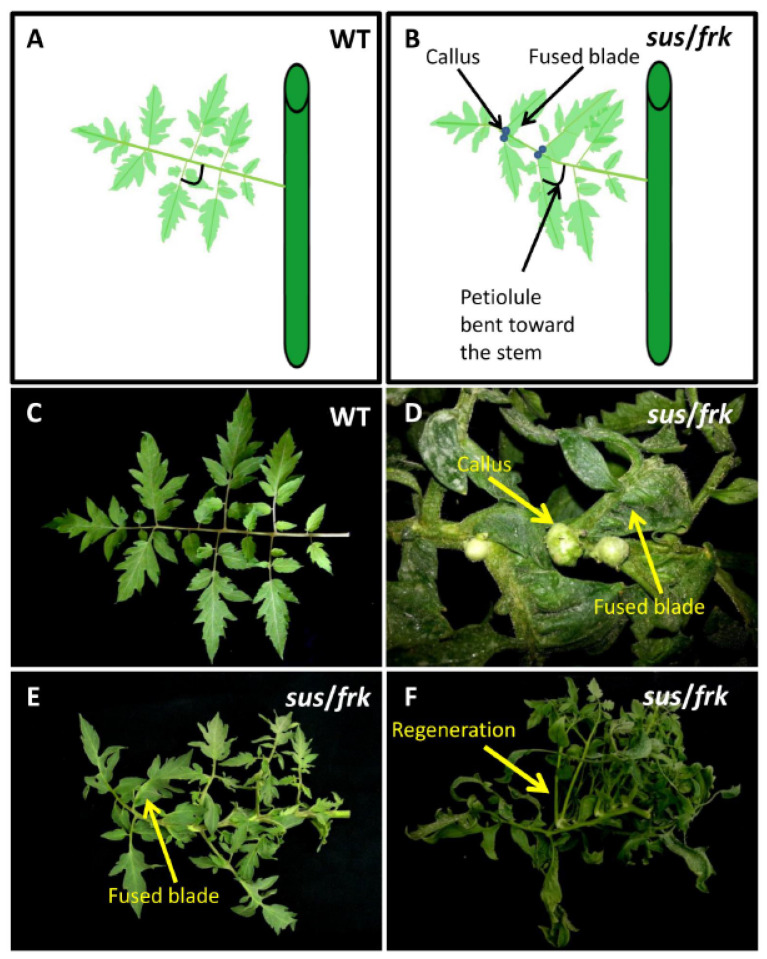
Morphological aberrations in *sus/frk* leaves. Schematic representations of (**A**) WT and (**B**) *sus*/*frk* leaves depicting the formation of callus tissue in the axil of the leaflet on the adaxial side of the leaf, the fused blade, and the petiolules bent toward the stem. (**C**) WT leaf. (**D**) A *sus*/*frk* leaf with a fused blade and callus (arrows). (**E**) A *sus*/*frk* leaf with a fused blade (arrow). Notice the bent petiolules. (**F**) In this *sus*/*frk* leaf, callus bulks transformed into shoot meristems and developed into new branches.

**Figure 6:**
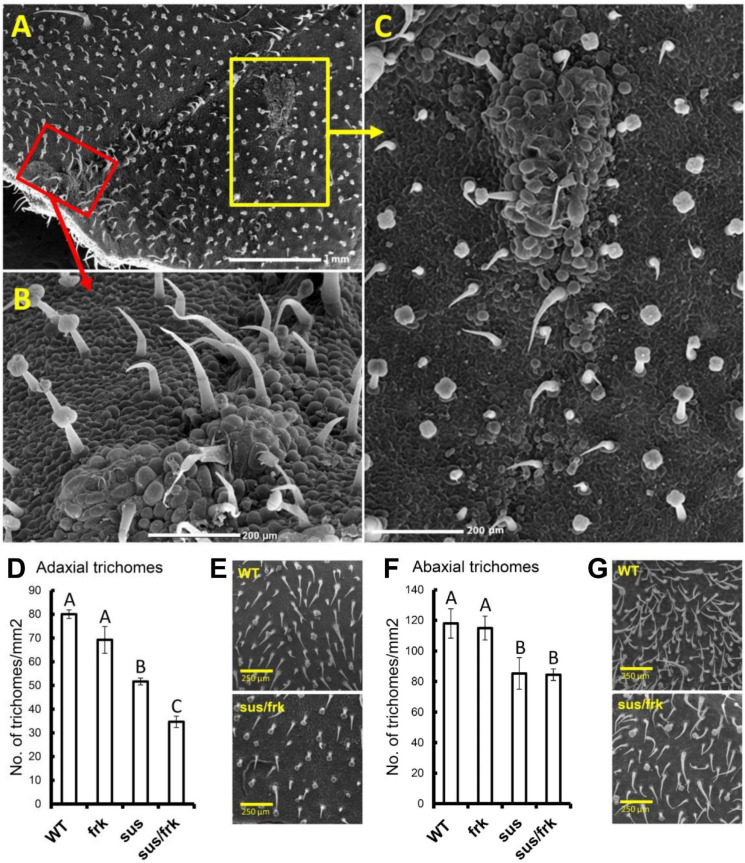
Formation of callus tissue on the adaxial blade of a *sus*/*frk* leaf and the *sus*/*frk* leaves showed reduced trichome density on both their adaxial and abaxial sides. (**A**) Adaxial blade of a *sus*/*frk* leaf. (**B**) Enlargement of the area indicated by the red box in (**A**). Notice the callus tissue. (**C**) Enlargement of the area indicated by the yellow box in (**A**). The arrows point to callus formation. (**D**) Total trichome density on the adaxial side of the blade among *frk*, *sus* and *sus*/*frk* plants, compared to the WT. (**E**) SEM images of the WT and *sus*/*frk* adaxial leaf surfaces. (**F**) Total trichome density on the abaxial side of the blade in *frk*, *sus* and *sus*/*frk* plants, compared to the WT. (**G**) SEM images of WT and *sus*/*frk* abaxial leaf surfaces. Error bars indicate the standard error (*n* ≥ 4). Different letters indicate a significant difference (*t*-test, *p* < 0.05).

Suppression of *SUSU1,3&4* affected the leaf morphology of the *sus* parent line [12] and similar effects were observed in the *sus*/*frk* plants. The leaf petiolules of the *sus*/*frk* line bent backward toward the stem and the leaves exhibited ectopic blade outgrowth, particularly in the region of the leaflets and lobes, proximal to both the petiole and the rachis (Figure 5B,D,E). In addition, unlike the *sus* line, in the *sus*/*frk* line, callus tissue formed in the axil of the leaflet on the adaxial side of the leaf (Figure 5B,D). Some of this callus tissue transformed into a shoot meristem and developed into new branches (Figure 5F). SEM of the adaxial blade surface of the leaflet revealed that collections of callus cells also appeared on the surface of the leaflets localized above junctions between the veins of the leaflet (Figure 6A–C). SEM of the leaf surface also revealed a reduction in trichome density in the *sus* and *sus*/*frk* lines, on both the abaxial and adaxial sides of their leaves (Figure 6D–G). The reduction in the adaxial trichome density was more severe among the *sus*/*frk* plants than it was among the *sus* plants (Figure 6D,E). More glandular trichomes developed on the abaxial sides of the leaves of *sus*/*frk* plants, as compared to the WT; whereas the density of the glandular trichomes on the adaxial side did not differ from that observed for the WT (Figure S2).

### 3.4. The sus/frk Line Exhibited Abnormalities in Its Vascular Tissue Anatomy

**Figure 7:**
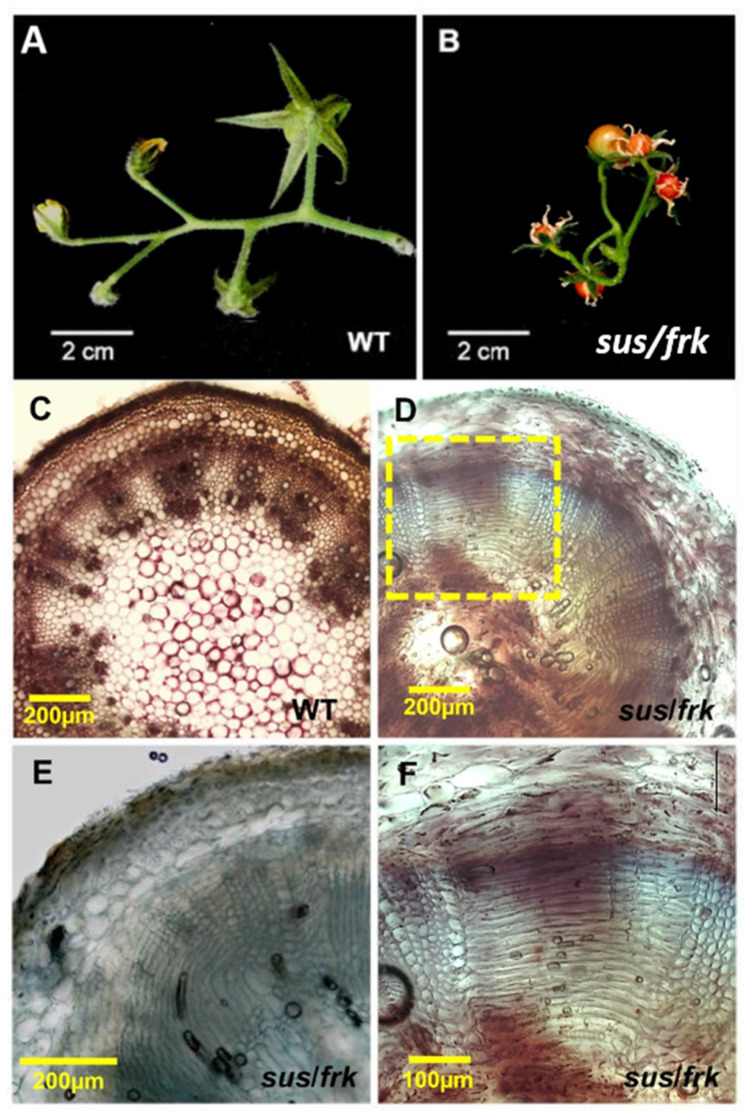
The *sus*/*frk* line exhibited distinct inflorescence architecture and disturbed directionality of xylem and parenchyma cells. (**A**) WT inflorescence. (**B**) *sus*/*frk* inflorescence exhibiting altered peduncle phyllotaxis. (**C**) Cross-section of a WT peduncle. (**D**,**E**) Cross-section of a *sus*/*frk* peduncle in which the orientation of the parenchyma xylem cells was disturbed. (**F**) Enlargement of the xylem area is indicated by the yellow box in (**D**).

In addition to the altered leaf morphology, the *sus*/*frk* line exhibited distinct inflorescence architecture characterized by an altered arrangement of the pedicels on the peduncle. In *sus*/*frk* more than one pedicel emerged from the same point and the inflorescence was not normally oriented and exhibited altered phyllotaxis (Figure 4A and Figure 7B as compared to Figure 7A). Cross-sections of the *sus/frk* peduncle revealed the presence of altered oriented xylem parenchyma cells (Figure 7D–F).

**Figure 8:**
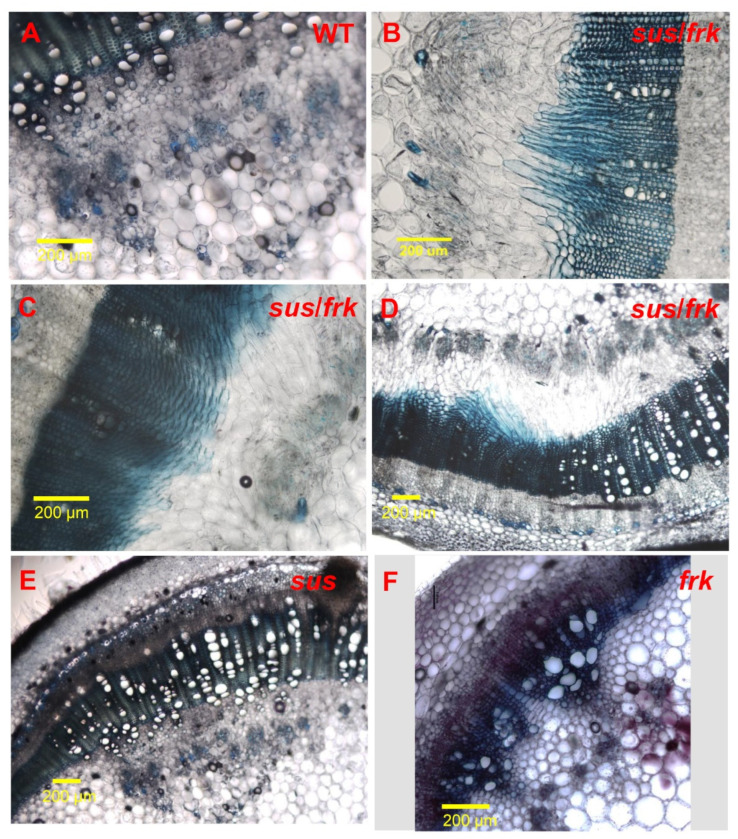
Abnormalities in the vascular tissue of the *sus*/*frk* stem. (**A**) Cross-section of a WT stem. (**B**–**D**) Cross-sections of *sus*/*frk* stems with disoriented xylem parenchyma cells. (**E**) Cross-section of a *sus* stem. (**F**) Cross-section of a *frk* stem.

The altered orientation of the xylem cells in the peduncle prompted us to examine the anatomy of the stem vascular tissue of the *sus*/*frk* plants. While the vascular-tissue anatomy of the *sus* plants was similar to that of the WT plants (Figure 8E; [12]), the *frk* plants had reduced xylem area and distorted (squashed rather than round) xylem vessels (Figure 8F; [10,11]). While no distorted xylem vessels were observed in the *sus*/*frk* stems, occasionally altered directionality of the xylem parenchyma cells was observed (Figure 8B–D). This altered cell orientation in the vascular tissues is unique to the *sus*/*frk* plants [20].

### 3.5. SUS and FRK Co-Suppression Caused SAM Arrest around the Transition to Flowering

**Figure 9:**
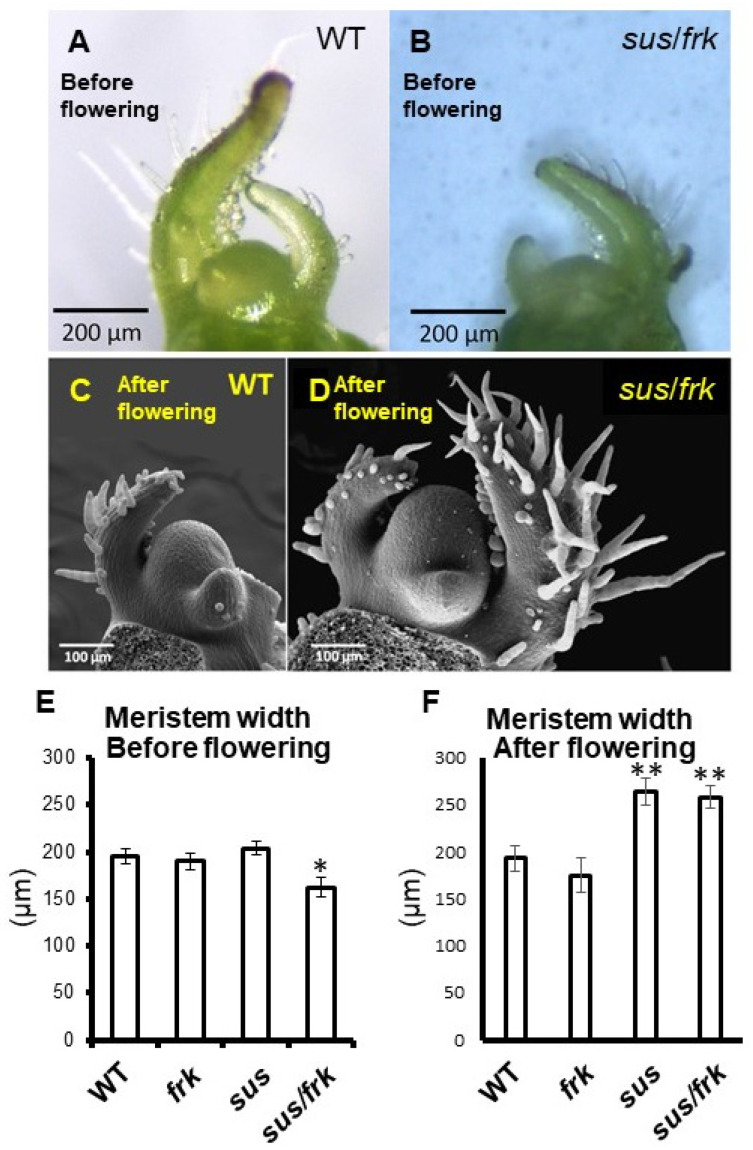
At an early vegetative phase, the SAMs of *sus*/*frk* are narrower than WT SAMs, but the SAMs of *sus*/*frk* and *sus* are larger after the transition to flowering. (**A**) Early vegetative SAM of a WT seedling. (**B**) Early vegetative SAM of a *sus*/*frk* seedling. (**C**) WT apical meristem (sympodial shoot meristem) after the transition to flowering and (**D**) *sus*/*frk* apical meristem after the transition to flowering. (**E**) Widths of the early vegetative SAMs of *frk*, *sus* and *sus*/*frk*, as compared to the WT. Data points are means ± SE (*n* ≥ 4). (**F**) Widths of the SAMs of *frk*, *sus* and *sus*/*frk*, as compared to those of the WT after the transition to flowering. Data points are means ± SE (*n* = 5). An asterisk indicates a significant difference relative to the WT (* *p* < 0.05; ** *p* < 0.01).

Another unique phenotype of *sus*/*frk* plants is the occurrence of SAM arrest after the appearance of about 10 leaves in approximately 30% of the plants (10.25 ± 1.44). The fact that this arrest occurs after the appearance of the 10th leaf suggests that it might be related to the transition of the plants to flowering, a transition stage of the shoot apical meristem that happens after the appearance of the 10th–11th leaf in WT plants of this genotype. Visual analysis of the SAM during the early vegetative phase revealed that the *sus*/*frk* SAMs were narrower than those of the WT and the parent *sus* and *frk* lines (Figure 9A,B,E and Figure S3A,B). However, after the transition to flowering, the SAMs of the *sus/frk* and *sus* plants were enlarged (Figure 9C,D,F and Figure S3D).

### 3.6. The SAMs of the sus/frk Plants Exhibited an Altered Auxin Response

The aberrations in leaf morphology and inflorescence architecture and the size and arrest of the SAM may indicate some alteration in auxin transport and signaling. Altered polar auxin transport has already been demonstrated for the *sus* line using the PIN1 promoter fused to the GFP reporter protein [12]. To gain insights into auxin-signaling and the auxin response in the *sus*/*frk* line, we crossed the plants with a tomato line expressing the VENUS reporter protein under the synthetic auxin-response promoter DR5 (DR5::VENUS; [18]). We then viewed the expression patterns of DR5::VENUS in F3 homozygous plants under a confocal microscope.

**Figure 10:**
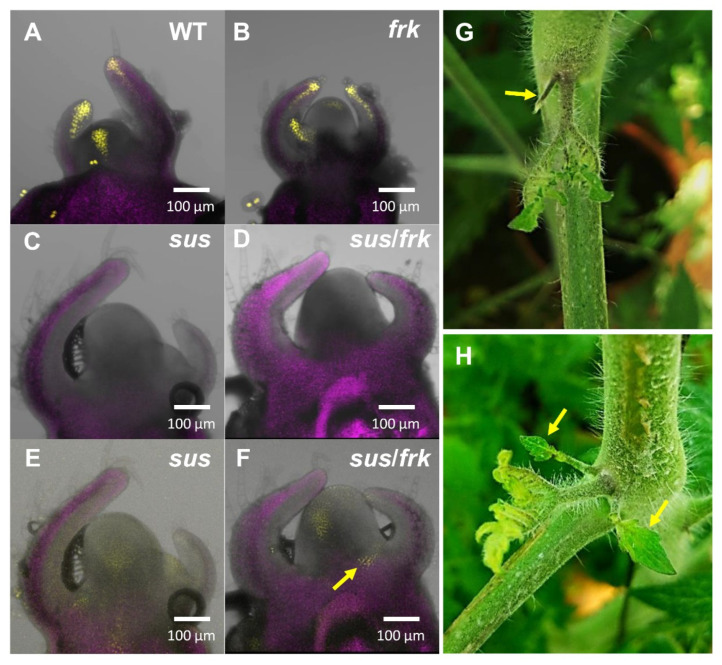
*sus*/*frk* SAM exhibits an altered auxin response. (**A**–**F**) Confocal images of SAMs of flowering WT, *frk*, *sus* and *sus*/*frk* plants expressing DR5::VENUS. All panels are merged images of white light, chlorophyll autofluorescence (stained magenta), and VENUS fluorescence (stained yellow). Exposure parameters in (**A**–**D**) are the same, while the exposure parameters in (**E**,**F**) are 50% bigger. (**A**) WT SAM. (**B**) *frk* SAM. (**C**) *sus* SAM. (**D**) *sus*/*frk* SAM. (**E**) The same *sus* SAM shown in (**C**) with the exposure parameters increased by 50%. (**F**) The same *sus*/*frk* SAM shown in (**D**) with the exposure parameters increased by 50%. (**G**) Pin leaf in *sus*/*frk* (arrow) that was formed in the same spot as the ectopic auxin maxima shown in (**F**) (arrow). (**H**) Formation of an extra leaf adjacent to a developing lateral branch in *sus*/*frk* (arrows), in the same spot as the ectopic auxin maxima shown in (**F**) (arrow).

While WT and *frk* plants showed normal DR5::VENUS expression patterns in their meristems and primordia (Figure 10A,B), neither the *sus* nor the *sus*/*frk* plants showed any fluorescent signal using the exact exposure parameters (Figure 10C,D). This implies that there are low levels of auxin in *sus* and *sus*/*frk* meristems and primordia. Only when the detection sensitivity was increased by 50% did the *sus* and *sus*/*frk* show a fluorescent signal, which was still weak compared to that observed for the WT and *frk* plants (Figure 10E,F). In addition, the *sus*/*frk* plants showed abnormal expression of DR5::VENUS of auxin maxima between the P2 primordia and the meristem (based on four out of six meristems; Figure 10F, arrows). Such unique auxin maxima between the leaf and the stem coincide with *sus*/*frk*’s frequent abnormal formation of pin-like leaves at the site of the auxiliary meristem (Figure 10G) or an extra leaf adjacent to the developing lateral branch (Figure 10H).

## 4. Discussion

### 4.1. Comparison of the Phenotypes Obtained from Co-Suppression of Both FRK and SUS with Those of the Parental Lines

It was over two decades ago that researchers first suggested that SuSy and *FRK2* work together to adjust carbon flux into certain metabolic pathways in different sink tissues [10,14,21]. Yet, the current study is the first experimental work to examine the developmental functioning of *SUS1,2&3* and *FRK2*, by using tomato plants with reduced expression of these genes. The phenotypes of the offspring from the cross between *frk* and *sus* plants are closer to that of the *sus* plants rather than that of the *frk* plants. The absence of the growth inhibition of the *frk* line in the *sus*/*frk* homozygote plants is probably due to the reduced suppression of *FRK2* in the *sus*/*frk* plants, as compared to the *frk* plants (Figure 3A). Reduced suppression of *FRK2*, from near-complete suppression to about 60% has been described previously for *frk* plants and attributed to the reduction in the copy number of the *FRK2*-antisense transgene, from three copies to two [15]. It has previously been shown that only near-complete suppression of *FRK2* is accompanied by growth inhibition [10,11]. We speculate that the original *sus*/*frk* plants that remained dwarf-like and failed to develop any further (Figure 1) maintained a high level of *FRK2* suppression; whereas the plants that eventually grew were those with reduced suppression of *FRK2*. These original sus/frk lines that failed to grow eventually died and, therefore, the suppression level of *FRK2* in those plants could not be verified. Additional work will be needed to clarify this speculation by generating additional F1 seedlings and linking their phenotype to their *FRK2* expression level. Another strategy can be metabolic rescue (by sugar supplements) of highly suppressed F1 seedlings.

The *sus*/*frk* plants that managed to grow still had reduced expression of both *SUS1,3&4* and *FRK2* genes and showed more pronounced phenotypes than the parental lines, including further reductions in seed weight and the germination rate and shorter hypocotyls within their embryos. In addition, the *sus*/*frk* plants exhibited unique phenotypes that are completely new and have not been observed in either of the parental lines, allowing us to study the combined roles of these enzymes. These phenotypes include meristem arrest around the transition to flowering, altered inflorescence phyllotaxis, the formation of abnormal lateral branches and callus tissue, shoot formation on the axil of the adaxial side of mature leaves, and the altered orientation of xylem cells in the stem and peduncle.

### 4.2. The Importance of SuSy and FRK for SAM Functioning during Flowering

There was little evidence that SuSy is an important factor for meristem development, or that it plays a role in the transition to flowering. Based on RNA-seq data obtained by Park et al. (2012) [22], it was previously shown that out of the six *SUS* genes in tomatoes, only *SlSUS1,3&4* are expressed in the meristems and primordia throughout plant development [12]. *SlSUS3* expression is especially high in transition meristem and in sympodial inflorescence meristems; whereas *SlSUS1* expression appears to be relatively high in transition meristem and highest in the flower meristem. *SlSUS4* expression appears to be relatively constant in both meristems and in primordia at all developmental stages [12]. *SlSUS1* transcript was also localized to very young primordia at their initiation stage using in situ hybridization with a *SUS* antisense probe [23].

In addition, Pien et al. (2001) [23] reported that *SlSUS1* transcript levels increased in response to sucrose and fructose treatments. It should be mentioned that while the Pien et al. (2001) [23] study referred to the *SlSUS1* transcript as *SUS4*, the mRNA sequence used to create the probe for *SlSUS1* (GenBank: L19762) corresponds to and is 100% identical to the *SUS* gene located in chromosome 12 that was defined as *SlSUS1* [12]. Lastly, the over-expression of Arabidopsis *SUS* genes in tobacco (*Nicotiana tabacum*) plants resulted in bifurcated stems in about 30% of the plants, as compared to none in the WT [24], indicating that SuSy may be involved in this SAM split event. In conclusion, the expression patterns of tomato *SUS* genes fit well with the SAM and primordia phenotypes of the *sus* line, in which co-suppression of all three genes causes abnormal leaf development [12].

*FRK1,2&3* genes are also expressed in the SAM, as can be seen from the RNA-seq data for that tissue (Figure S4). *FRK2&3* were expressed at a higher level than *FRK1* and the expression level of *FRK2* increased as the meristem matured. That observation fits with the meristematic aberrations of the double *sus/frk* mutant, namely, smaller meristems during the vegetative growth and meristem arrest around the transition to flowering.

### 4.3. SuSy and FRK Interact with Auxin Pathways

Auxin is a major plant hormone involved in many developmental processes such as meristematic leaf initiation and development, the differentiation of vascular tissues, and embryo development. It is still not clear how suppression of *SUS* and *FRK* affects the auxin response. One possible explanation could be that the reduced expression of these genes in the SAM alters the concentrations of the soluble sugars sucrose, glucose, and fructose, which in turn affect auxin-related gene expression, possibly affecting auxin synthesis, transport, or signaling. A growing line of evidence suggests that sugars may be important for auxin synthesis, transport, and signaling. Glucose was found to induce auxin biosynthesis in Arabidopsis via several tryptophan-dependent biosynthetic pathways, in a manner that is at least partly mediated by PHYTOCHROME-INTERACTING FACTORS (PIFs; [25,26,27,28,29,30]). Glucose alone could transcriptionally regulate 62% of the genes that are also regulated by auxin in Arabidopsis seedlings, including genes related to auxin syntheses like YUCCAs, auxin transport-like PIN1, auxin receptors, auxin-response factors (ARFs), auxIAAs and other auxin-induced genes [31]. Lastly, our previous work suggested that SuSy may be important for auxin signaling and transport based on *sus* plants that had altered leaf morphology, distorted distribution of PIN1-GFP localization in leaflet primordia, and changes in relative expression levels of auxin-related genes in the shoot apex like JAG, PIN1 and IAA9 [12].

The results from the co-suppression of *SUS1,3&4* and *FRK2* in the current study reveal a new connection between SuSy, FRK, and auxin-related phenotypes exerted by more severe cotyledon morphology, abnormal inflorescence architecture, and vascular-cell orientation. The use of the DR5::VENUS reporter system revealed that SuSy may be more crucial than FRK for auxin signaling, as the intensity of the signal in the *sus* line was very low and similar to that observed in the *sus*/*frk* line; whereas, in this respect, the *frk* line was similar to the WT (Figure 10). Nonetheless, the additional auxin-related phenotypes observed in the double *sus*/*frk* line suggest that *FRK2* may also be important for auxin signaling and transport.

The larger SAMs observed in the *sus*/*frk* line (Figure 9D,F and Figure 10) may also suggest altered auxin signaling. Shi et al. (2018) [32] claimed that auxin level is negatively correlated with SAM size. Accordingly, the larger SAMs in the *sus*/*frk* line support the notion that *sus*/*frk* plants have lower levels of auxin. This is further supported by the reduced intensity of DR5::VENUS fluorescence in the SAMs of *sus*/*frk* plants. Another phenotype observed in the *sus*/*frk* line that may be related to auxin level is the formation of callus tissue on leaf blades and the formation of shoots on leaf axils. Auxins and cytokinins have been shown to act antagonistically to promote callus formation or root regeneration, which could be affected by the ratio between those two phytohormones [33]. The induction of callus formation suggests that these areas might have low auxin-to-cytokinin ratios, which may be a result of reduced auxin synthesis or interruptions in auxin flow.

The reduced adaxial and abaxial trichome density of *sus*/*frk* might also suggest a reduction in auxin signaling, as auxin-dependent transcriptional regulation has been shown to be involved in trichome initiation in tomato [34,35]. The reduction in trichome density was evident in the *sus* line, but the *sus*/*frk* line exhibited a greater reduction of trichome density on the adaxial side, the same side on which callus formed in the *sus*/*frk* line, but not in the *sus* line. Yet, since auxin levels in mature leaves are expected to be relatively low, the *sus*/*frk* line’s callus and shoot generation could reflect increased cytokinin levels or greater reduction of auxin in mature leaves, which would alter the balance between auxin and cytokinin.

### 4.4. SuSy and FRK Are Important for Vascular Development

SuSy activity and expression have often been found to be associated with vascular tissues, implying that this enzyme may play an important role in vascular development [36]. Yet, the tomato *sus* line in which *SUS1,3&4* genes were co-suppressed did not show any significant vascular abnormalities [12]. Similarly, Arabidopsis double- and quadruple-*SUS* T-DNA mutants did not exhibit any unusual vascular-related developmental phenotype [37,38].

Unlike SuSy, FRK was found to be important for vascular development. Suppression of *FRK2* in tomato plants decreases stem xylem area and leads to the formation of distorted xylem vessels with thin cell walls [10,11]. It has been assumed that the main effect of *FRK2* suppression is primarily metabolic. Specifically, reduced fructose metabolism has been shown to limit cambium cell proliferation and the cell-wall synthesis of xylem vessels [10,11]. However, the double *sus*/*frk* line revealed that SUS and FRK are necessary for the proper orientation of the xylem parenchyma cells (Figure 7 and Figure 8). According to the canalization hypothesis [39], auxin flow may be a major factor in the differentiation and patterning of the vascular tissues and the disorientation of xylem cells may imply interference with the basipetal auxin transport through the stems of the *sus*/*frk* plants. Yet, we failed to detect any DR5::VENUS signal in the stem that might have indicated any such interference, perhaps because the expression of DR5::VENUS in the stem was below the threshold level for detection.

### 4.5. Concluding Remarks

In recent years, substantial progress has been made in identifying the molecular mechanisms by which sugars may act as signaling molecules in plants, with most of that work done in Arabidopsis. Although it is still quite difficult to differentiate between metabolic effects and signaling functions, a few putative mechanisms have been suggested. In Arabidopsis, it was suggested that HXK1 may act as a glucose sensor that adjusts plant responses to glucose levels regardless of its activity [40,41] and FRUCTOSE-1,6-BISPHOSPHATASE (FBP) was found to be crucial for fructose-mediated signaling [42]. While no sucrose sensors have been characterized to date, it has been suggested that sucrose-signaling may be mediated by trehalose 6-phosphate (T6P), a molecule found in very low quantities in plants whose concentration correlates to sucrose levels and which has profound effects on plant growth and development [43]. SuSy and FRK have been considered to be involved in sucrose metabolism rather than sugar-sensing, which together adjust carbon flux in sink tissues. Yet, the co-suppression of these enzymes in tomato plants revealed an unexpected connection between *SUS1,3&4*, *FRK2*, and auxin with regard to meristematic function and vascular development.

## Data Availability

The raw data supporting the conclusions of this article will be made available by the authors, without undue reservation.

## Supplementary Materials

The following are available online at https://www.mdpi.com/article/10.3390/plants11081035/s1, Figure S1. One-week-old seedlings of WT, *sus*, and *frk* plants. Figure S2. The *sus*/*frk* leaves showed increased trichome density on their abaxial side. (A) Glandular trichome density on the adaxial side of the leaf blade in *frk*, *sus*, and *sus*/*frk* plants, compared to the WT. (B) Glandular trichome density on the abaxial side of the leaf blade in *frk*, *sus*, and *sus*/*frk*, as compared to the WT. Error bars indicate the standard error (*n* ≥ 4). Different letters indicate a significant difference (*t*-test, *p* < 0.05). Figure S3. Meristems of *sus* and *frk* plants before and after flowering (A) Early vegetative SAM of a *frk* seedling. (B) Early vegetative SAM of a sus seedling. (C) *frk* apical meristem (sympodial shoot meristem) after the transition to flowering and (D) sus apical meristem after the transition to flowering. Figure S4. Expression of the *FRK* genes during meristem maturation. Expression data of *FRK1,2&3* genes obtained from RNA sequencing performed on meristems of the cultivar M82 during meristem maturation (http://tomatolab.cshl.edu/efp/cgi-bin/efpWeb.cgi, accessed on 2 April 2019). Early vegetative meristem (EVM), middle vegetative meristem (MVM), late vegetative meristem (LVM), transition meristem (TM), sympodial inflorescence meristem (SIM), and flower meristem (FM) were analyzed. RPKM—reads per kilobase per million mapped reads.
