# Supplementary figures and images for: Sucrose Synthase and Fructokinase Are Required for Proper Meristematic and Vascular Development
Source: Plants (Basel). 2022 Apr 11;11(8):1035. doi: 10.3390/plants11081035 (PMC9025968; doi:10.3390/plants11081035)

Figure S1

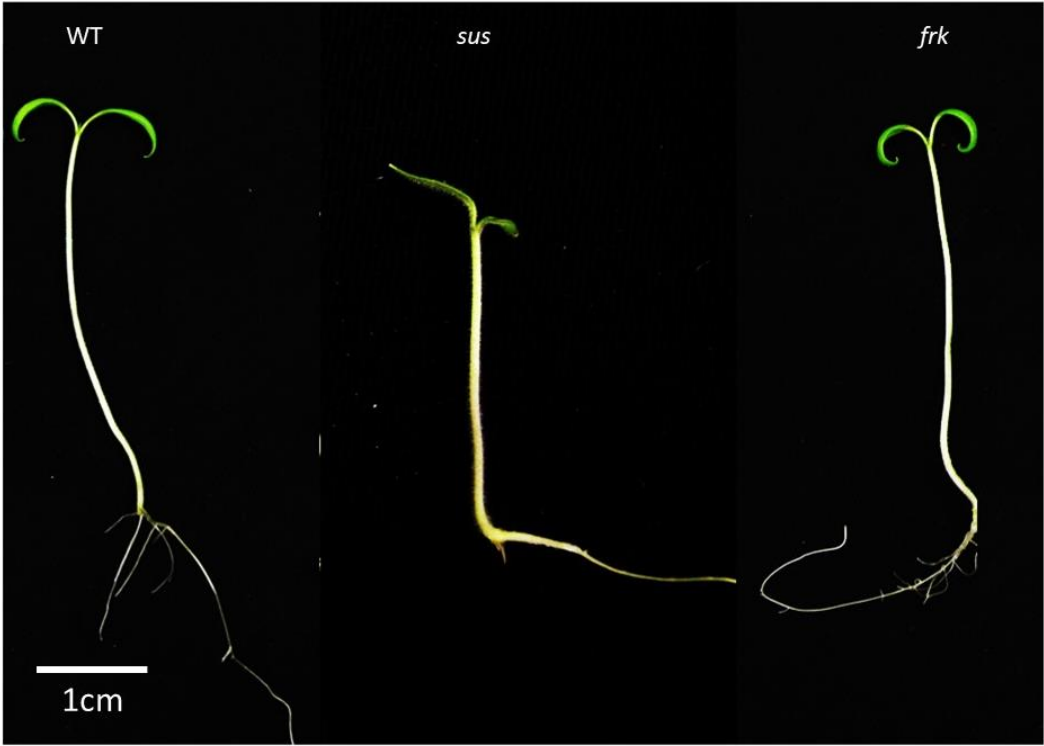

Figure S2

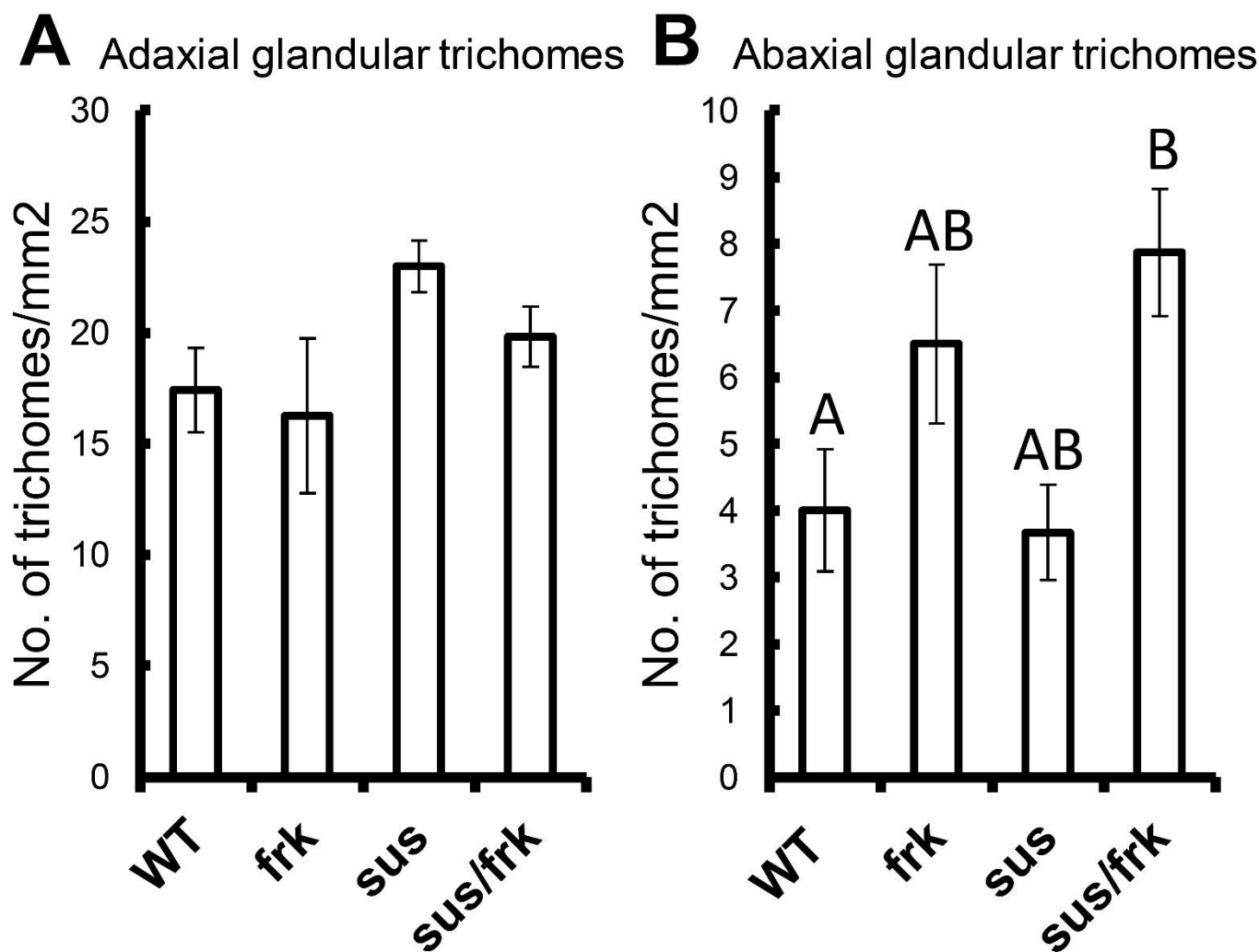

Figure S3

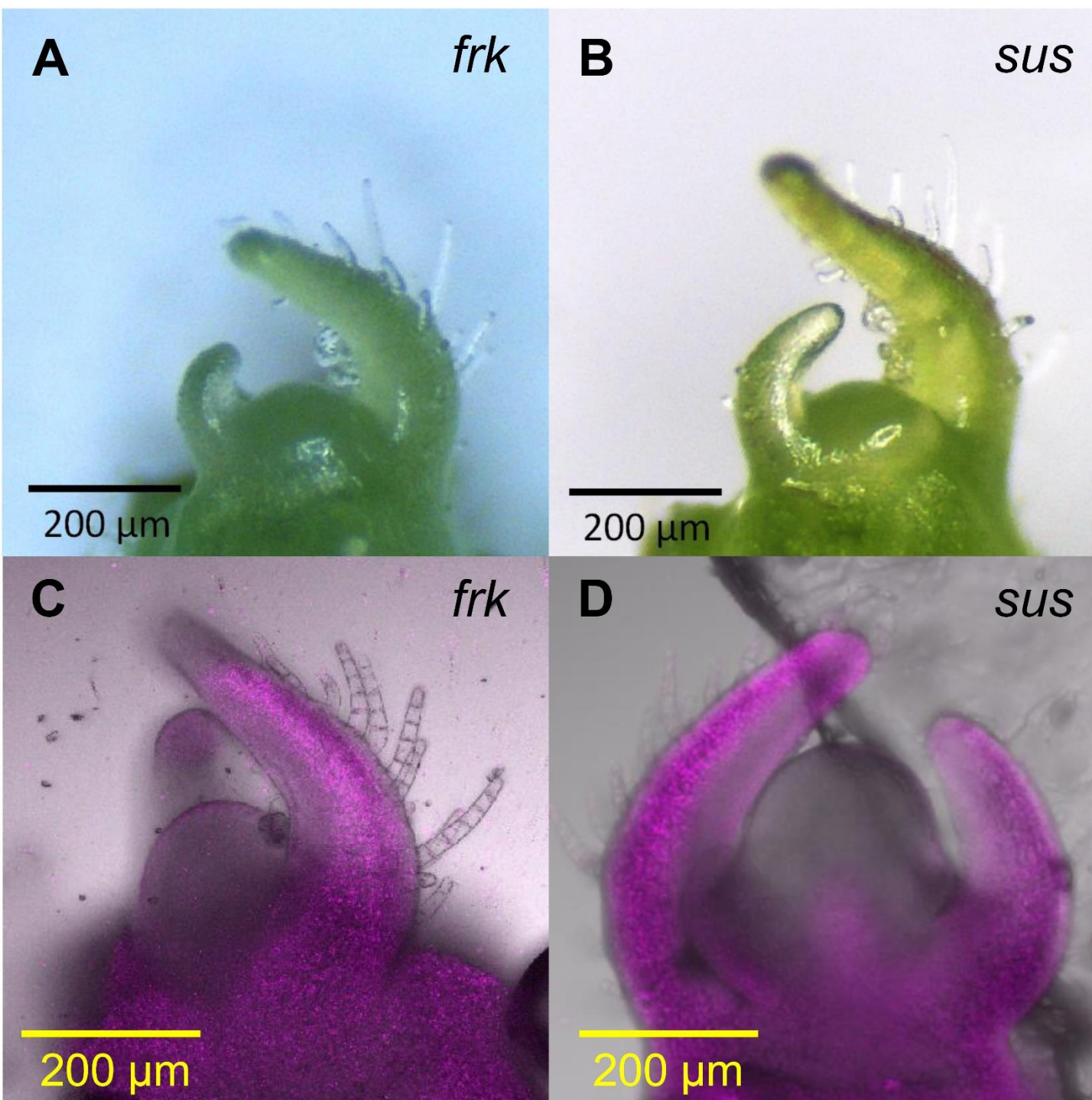

Figure S4

Meristems

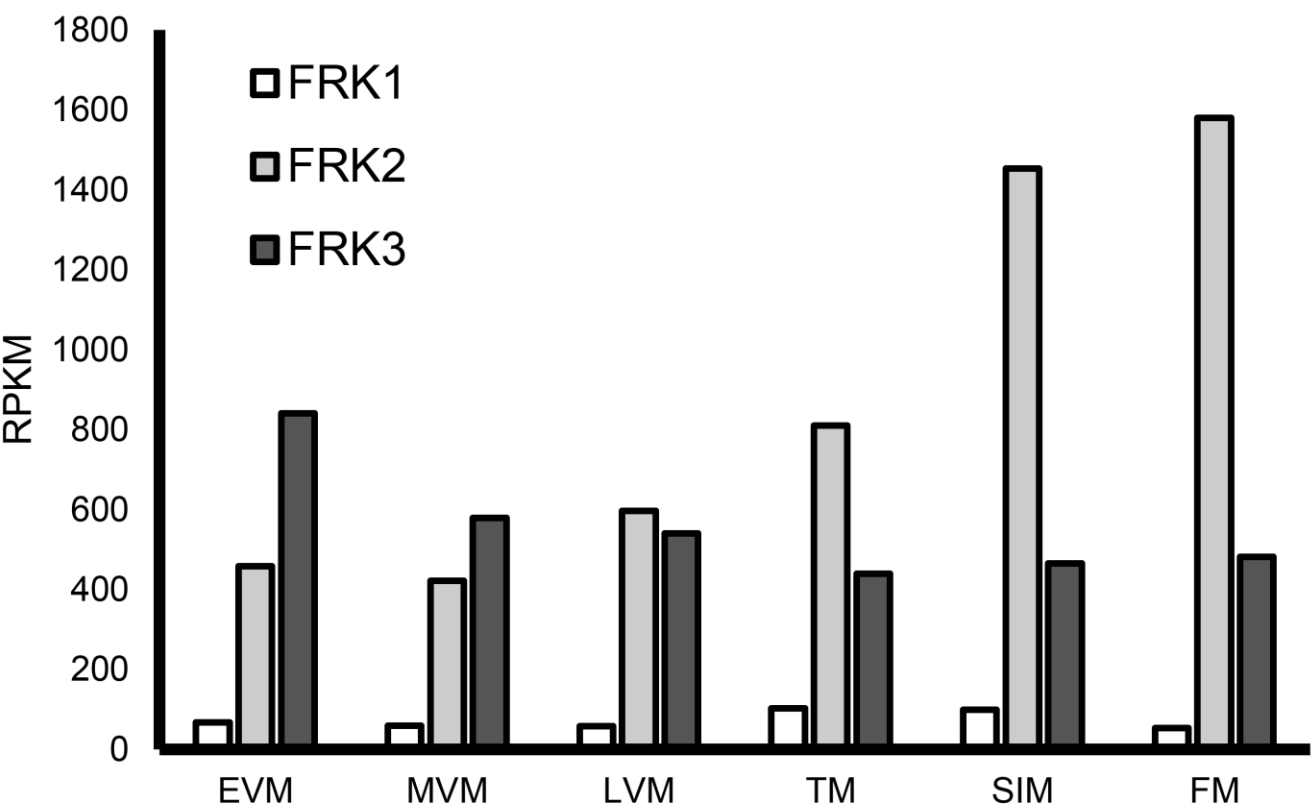

Supplement: Supplementary file 1 [file plants-11-01035-s001.zip › plants-1651833-supplementary.pdf]
